# Supplementary material for: SERINC5-Mediated Restriction of HIV-1 Infectivity Correlates with Resistance to Cholesterol Extraction but Not with Lipid Order of Viral Membrane
Source: Viruses. 2022 Jul 27;14(8):1636. doi: 10.3390/v14081636 (PMC9332783; doi:10.3390/v14081636)
Supplement: Supplementary file 1 [file viruses-14-01636-s001.zip › viruses-1748046-supplementary.pdf]

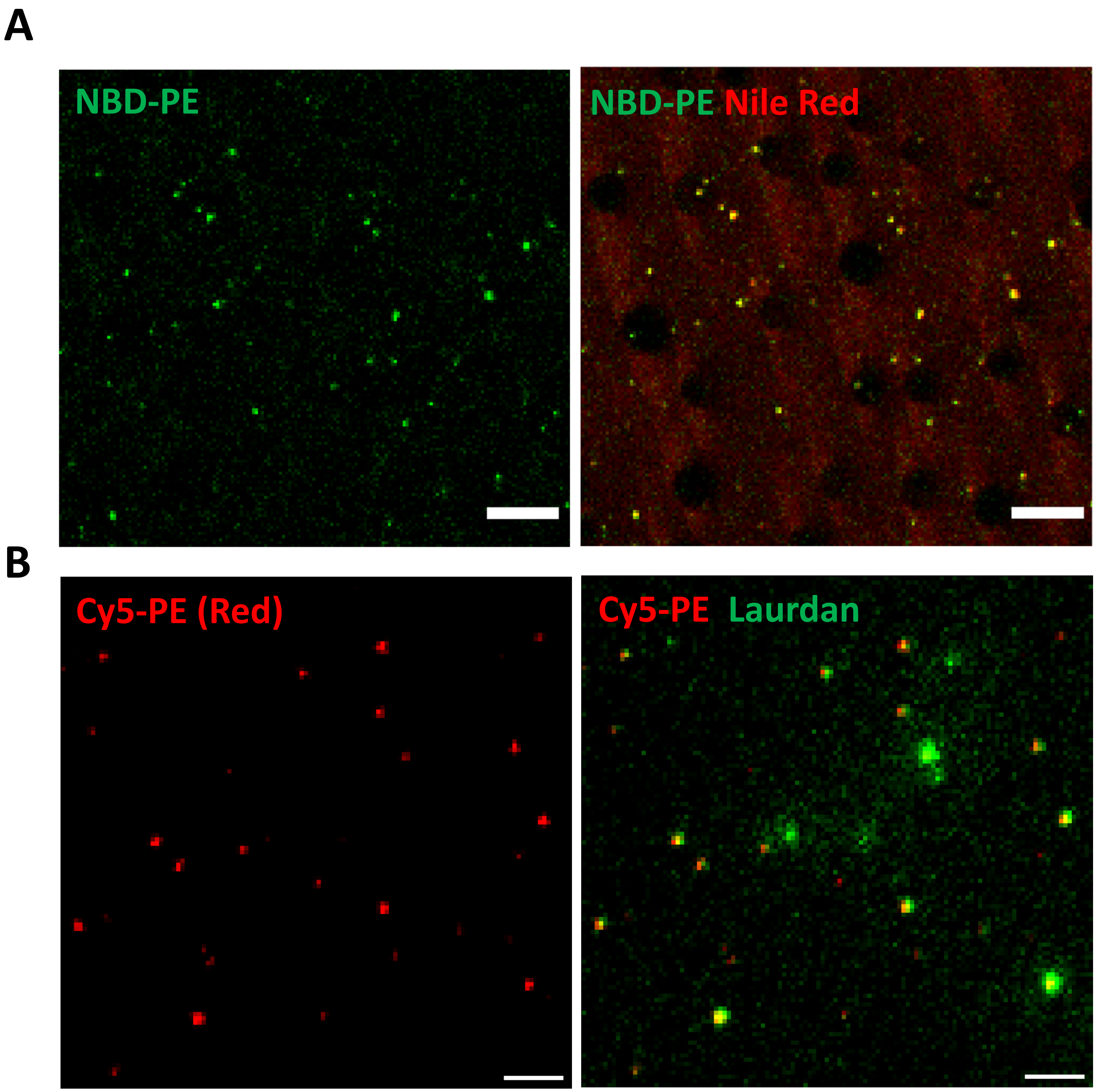

Figure S1. Lipid order measurements on single liposomes. Images of immobilized single NBD-PE-labeled liposomes stained with Nile Red (A) and Cy5-PE-labeled liposomes stained with Laurdan (B) Nile Red or Laurdan signals from liposomes were separated from background signal and dye aggregates based upon NBD-PE and Cy5-PE objects, respectively. Scale bar is 5 μm for both images.

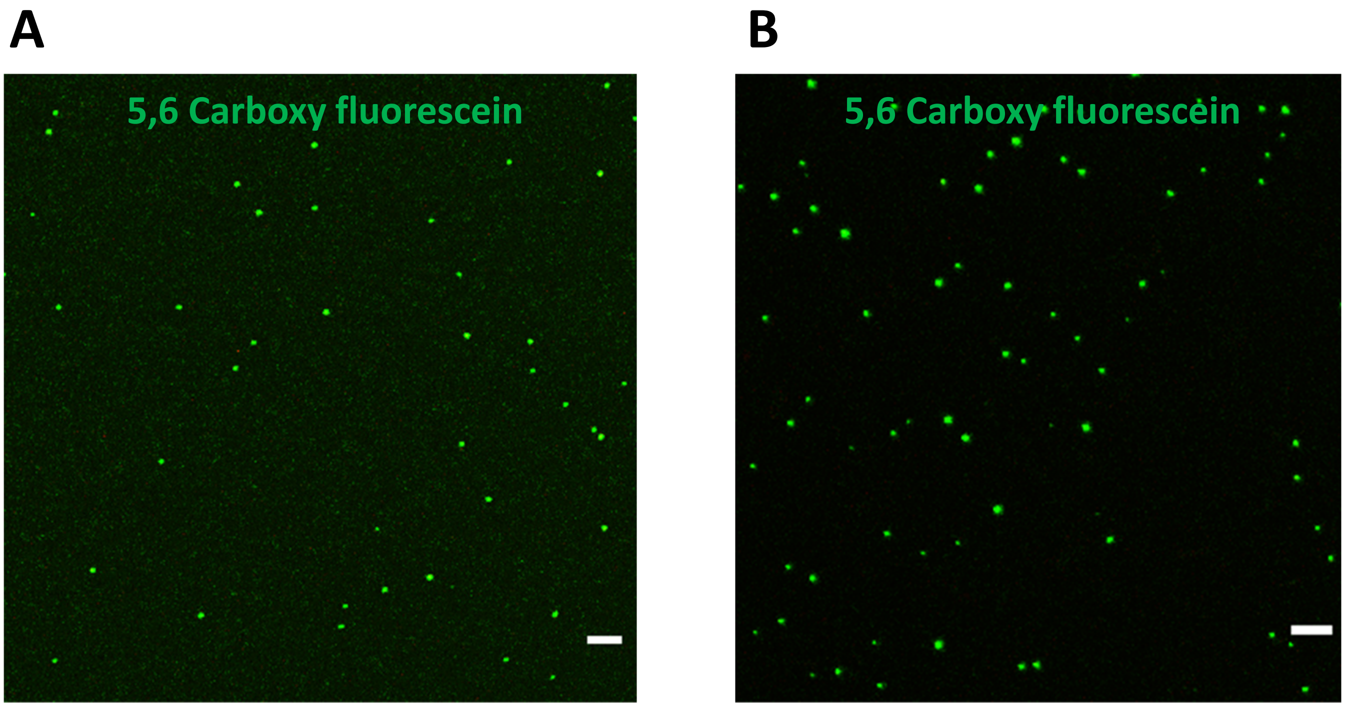

Figure S2. Dye loaded liposomes immobilized on the surface. Images of immobilized Lo liposomes (A) and Ld liposomes (B) on streptavidin coated surfaces. Liposomes were first loaded with soluble 5,6 carboxyfluorescein (1μM), then subjected to size exclusion chromatography to ensure removal of free-dye. The detection of distinct fluorescent puncta indicate that the immobilized liposomes are intact after immobilization. Scale bar (5 μm).

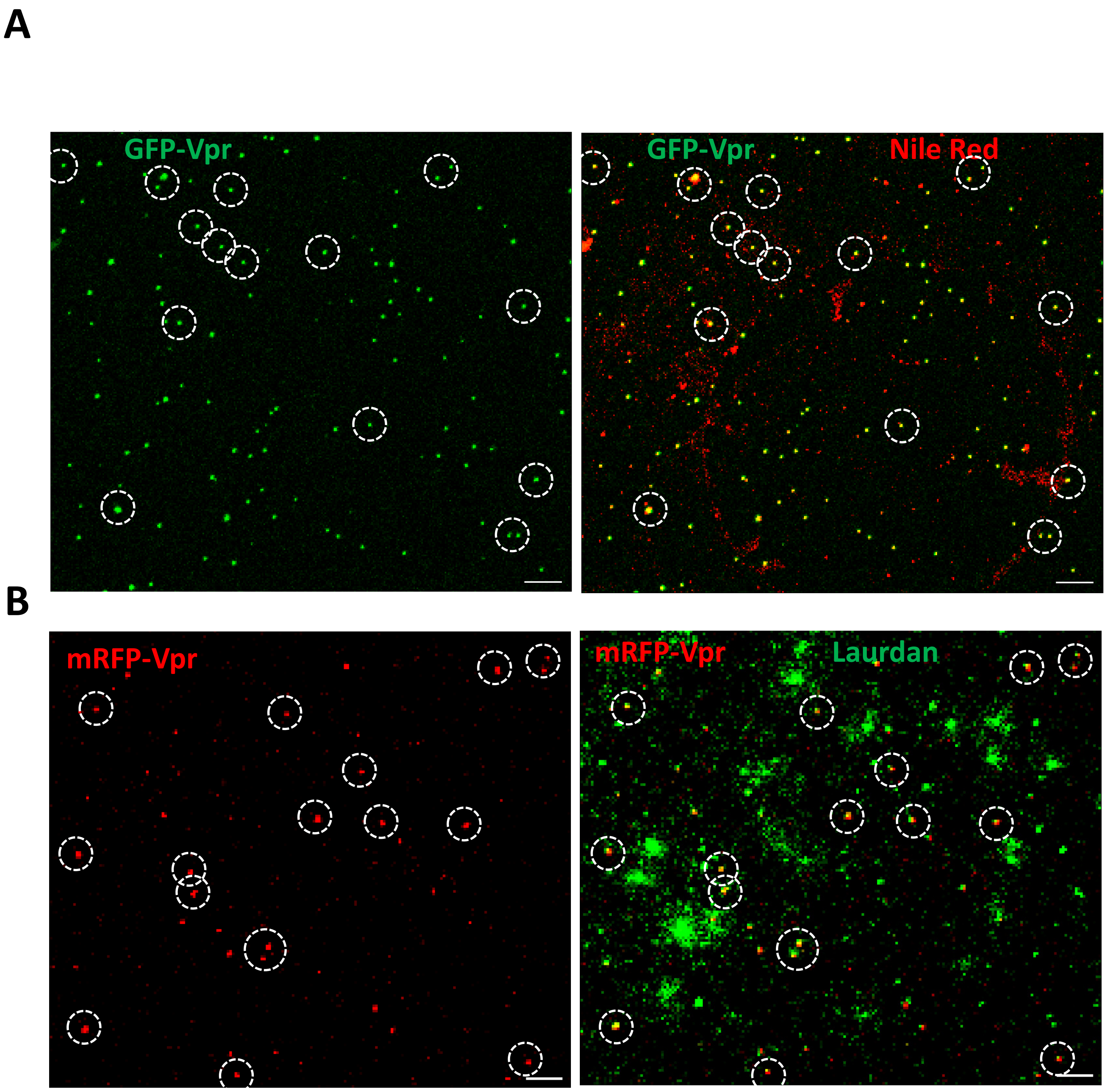

Figure S3. Lipid order measurements on single pseudoviruses. Images immobilized pseudoviruses labeled with GFP-Vpr (green) and stained with Nile Red (A) and pseudoviruses labeled with mRFP-Vpr (red) and stained with Laurdan (B). Localized Vpr coordinates (while circles) enable discrimination between background and dye aggregates and Nile Red and Laurdan signals from single viruses. Scale bar (5 μm) for both images.

A

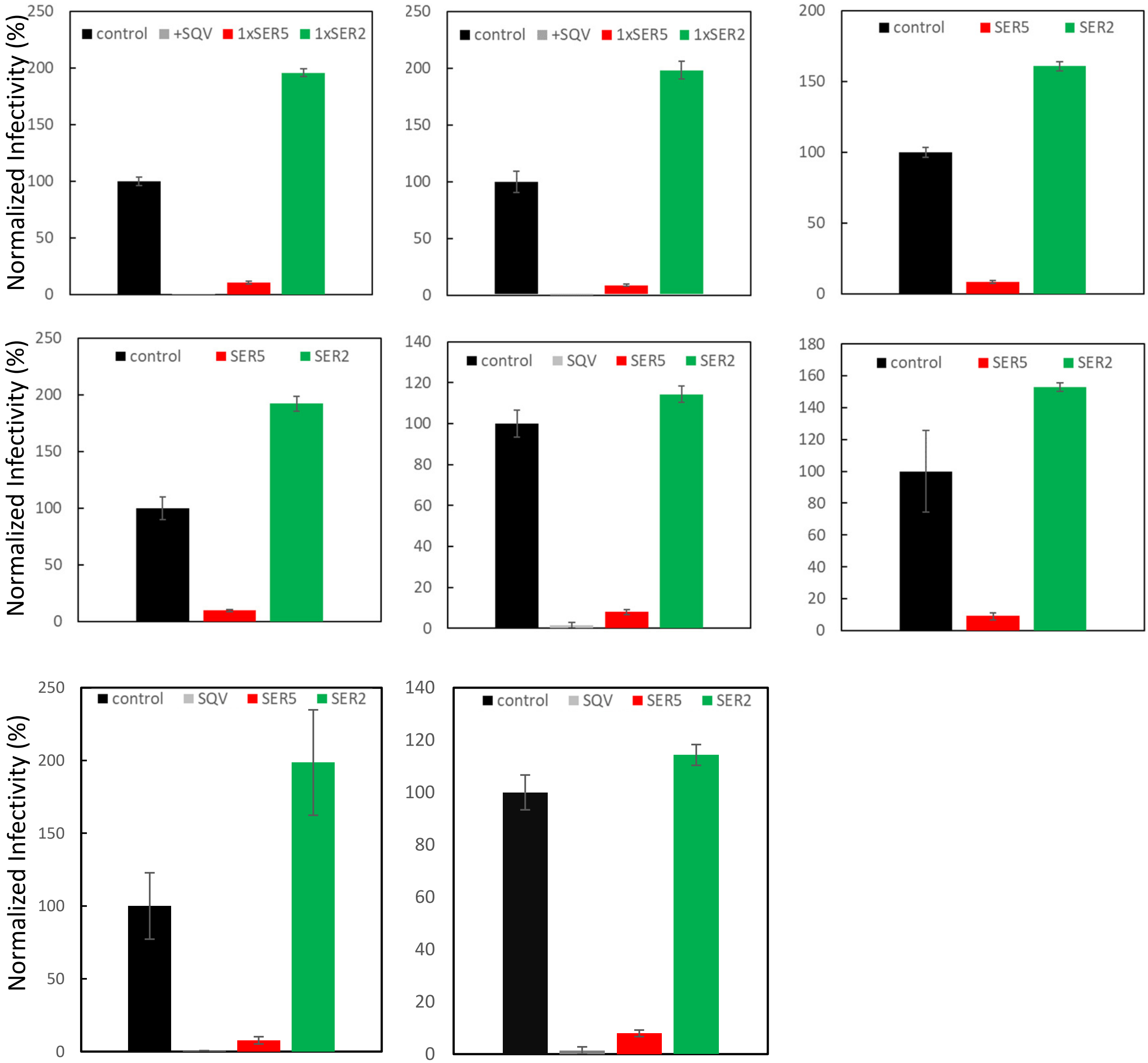

Figure S4. Characterization of pseudovirus preparations used for lipid order measurements and functional assays. (A) Pseudovirus preparations were characterized by measuring their relative infectivity (control as 100%) with a luciferase reporter assay and normalized by p24 of each virus.

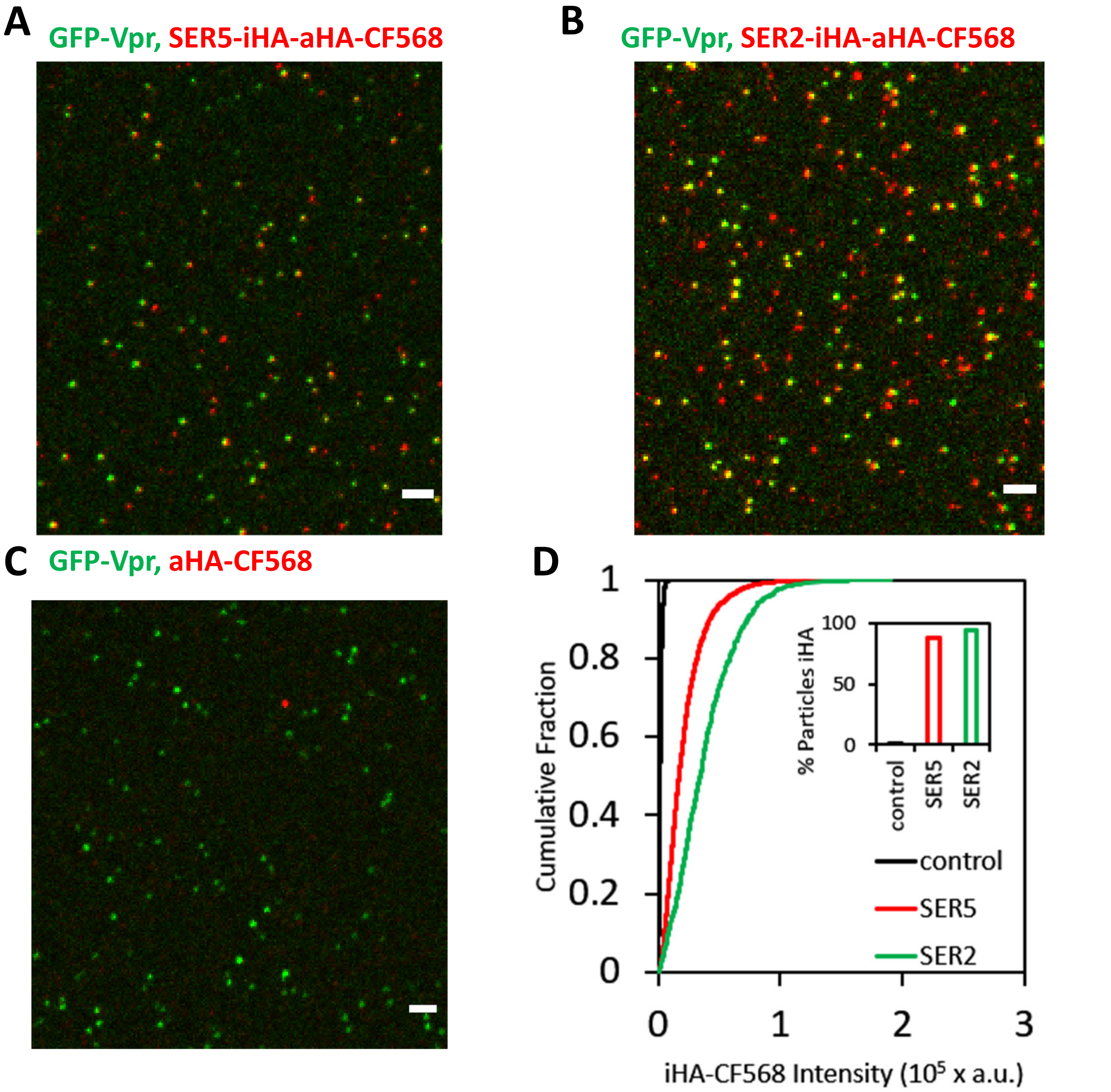

Figure S5. Characterization of SER5 and SER2 incorporation in GFP-Vpr pseudoviruses used for lipid order measurements and functional assays. SER5 (A), SER2 containing (B), or control (C) viruses were attached to coverslips, fixed and incubated with anti-HA (SERINC-iHA) antibody, followed by staining with goat anti-mouse CF568 antibody. (D) CF568 fluorescence signals from GFP coordinates were background subtracted and plotted as cumulative fraction. *Inset*: Percent colocalization of CF568 signals and GFP positive particles. Scale bar: 5 µm.

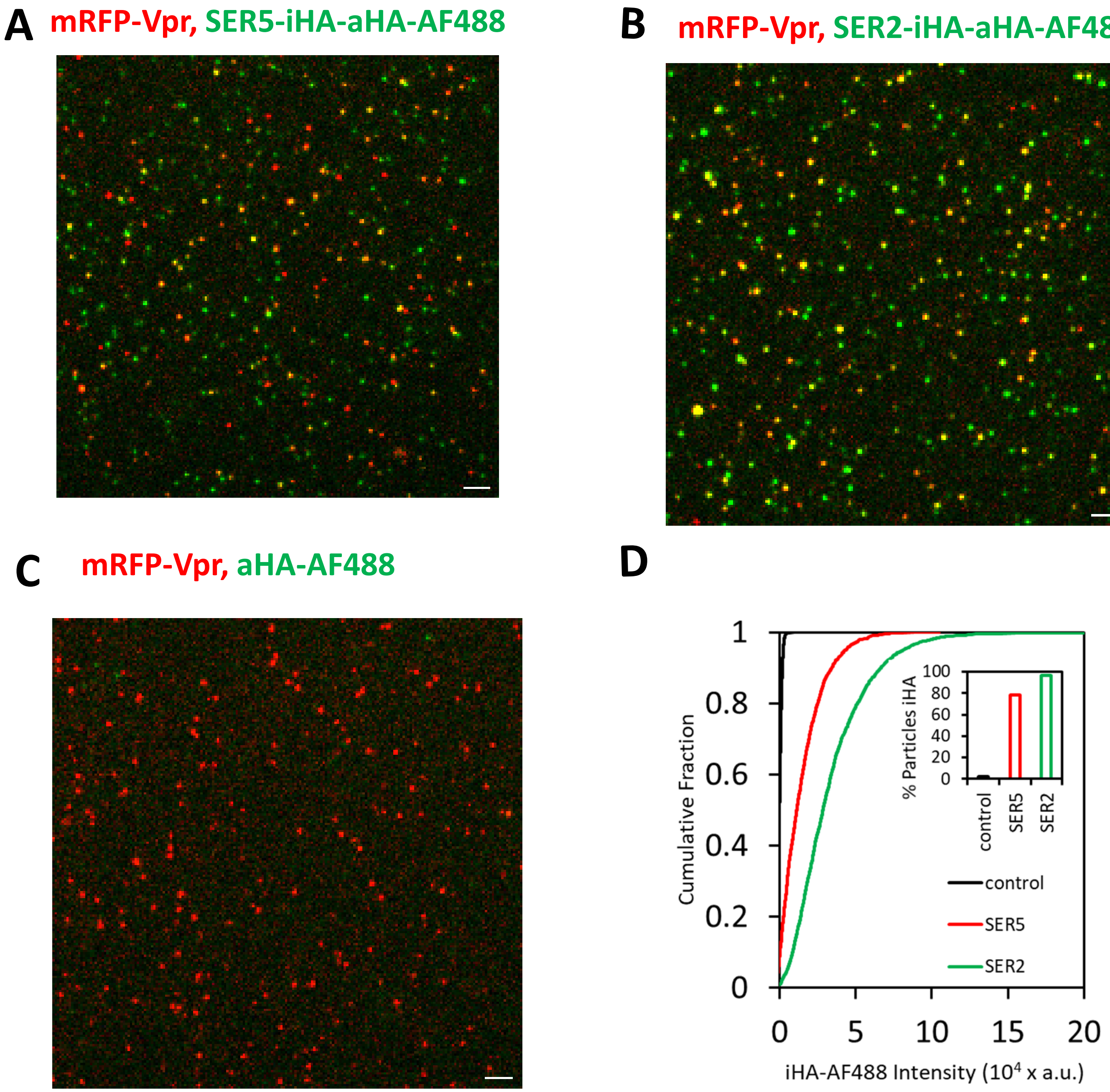

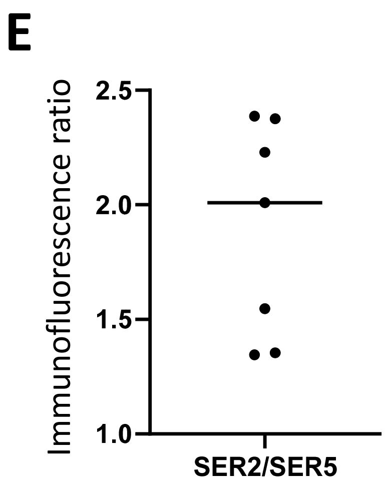

Figure S6. Characterization of SER5 and SER2 incorporation in mRFP-Vpr pseudoviruses used for lipid order measurements and functional assays. SER5 (A), SER2 containing (B), or control (C) viruses were attached to coverslips, fixed and incubated with anti-HA (SERINC-iHA) antibody, followed by staining with anti-mouse-AF488 antibody. (D) AF488 fluorescence signals from mRFP coordinates were background subtracted and plotted as cumulative fraction. *Inset*: Percent colocalization of AF488 signals and mRFP positive particles. (E) Immunofluorescence signals of SERINCs-iHA staining determine SER2/SER5 ratio of each pseudovirus preparations, including mRFP-Vpr and GFP-Vpr pseudoviruses (see Suppl. Fig. S6) prepared for Laurdan and NR staining, respectively. Scale bar: 5 µm.

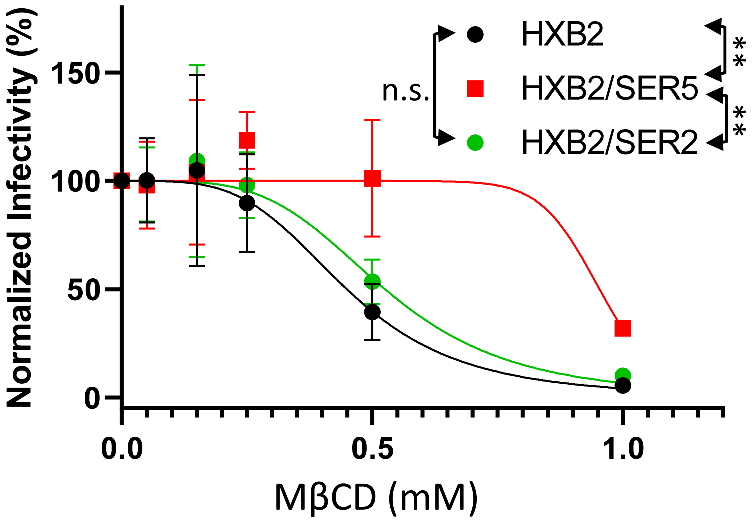

Figure S7. Inhibition of infectivity assays with virus cholesterol depletion by MβCD in solution. After viruses were treated with MβCD in solution, viruses were immobilized on a PDL-coated 96-well plate, washed by PBS++ and overlayed with TZM-bl cells. 2 independent virus preparations were treated with MβCD and compared by 2-way ANOVA repeated measures, applied to the descending part of infectivity curves (0.25-1mM MβCD). Infectivity data were normalized by untreated viruses and fitted by Hill-Langmuir equation. n.s., $p>0.05$; **, $0.01>p>0.001$.

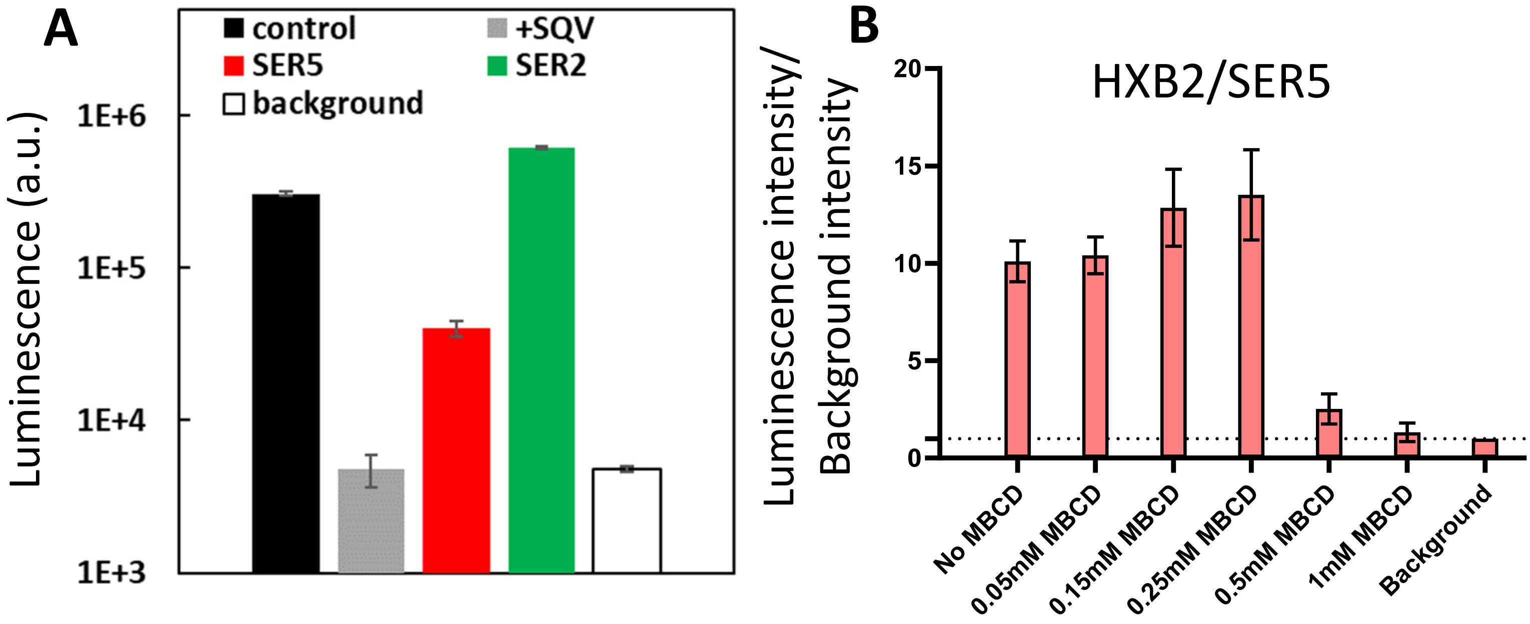

Figure S8. Dynamic range of infectivity assays. (A) A representative comparison of luminescence reading between control, +SQV (immature), SER5- and SER2-containing pseudoviruses, along with background (uninfected cells) in a 96-well plate. (B) Representative, raw (without background subtraction) luminescence intensities from SER5-containing pseudoviruses normalized to the no-virus background across [MβCD] demonstrates the dynamic range of the luciferase reporter assay at lower luminescence levels. Error bar: Standard deviation of 4 wells in each condition. Dotted line: Background level.

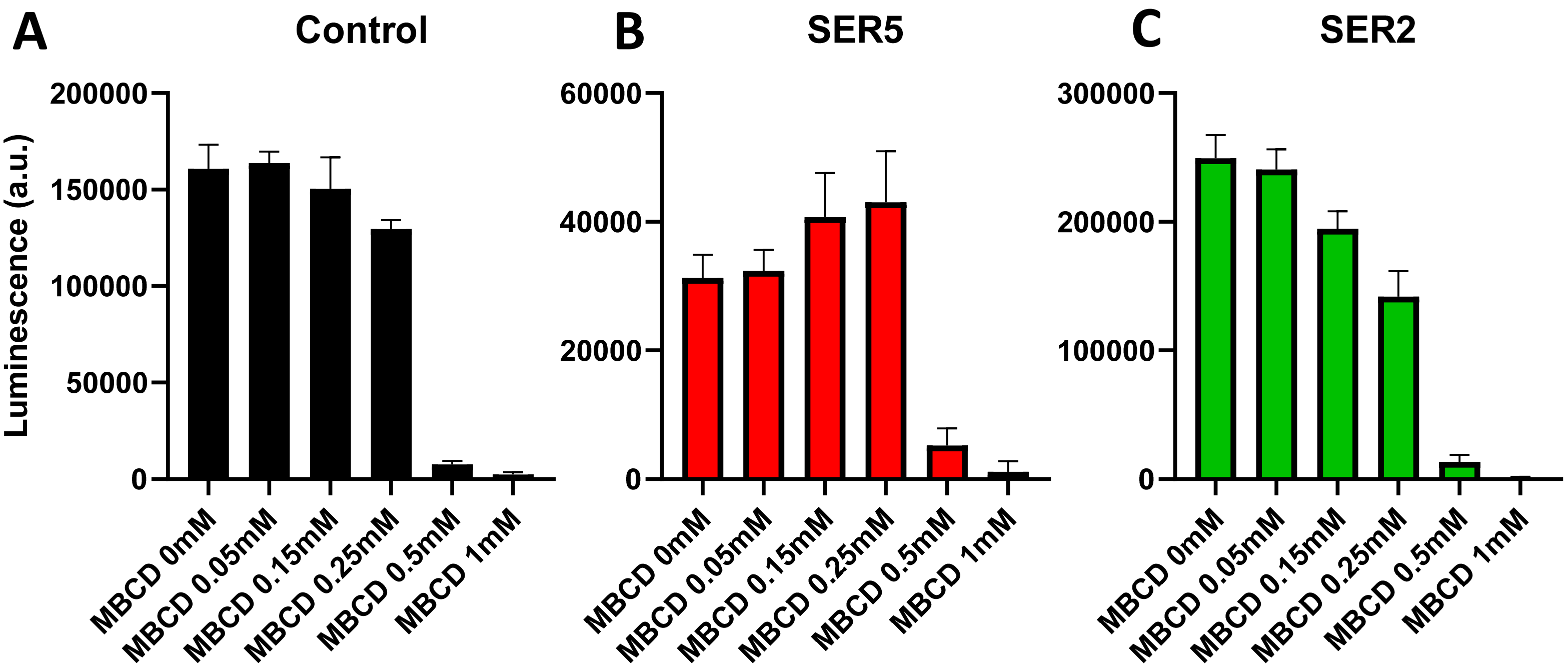

Figure S9. Luminescence data without normalization from luciferase reporter assay on viruses treated with MβCD. Background-subtracted luminescence intensity of a representative experiment obtained from luciferase reporter assay from 4 wells containing control (A), SER2 containing (B) or SER5 containing (C) pseudoviruses immobilized on a PDL-coated 96-well plate. Viruses were washed with PBS++ and incubated with TZM-bl cells for at least 36 hours. Error bars are standard deviations of 4 replicates in each condition.

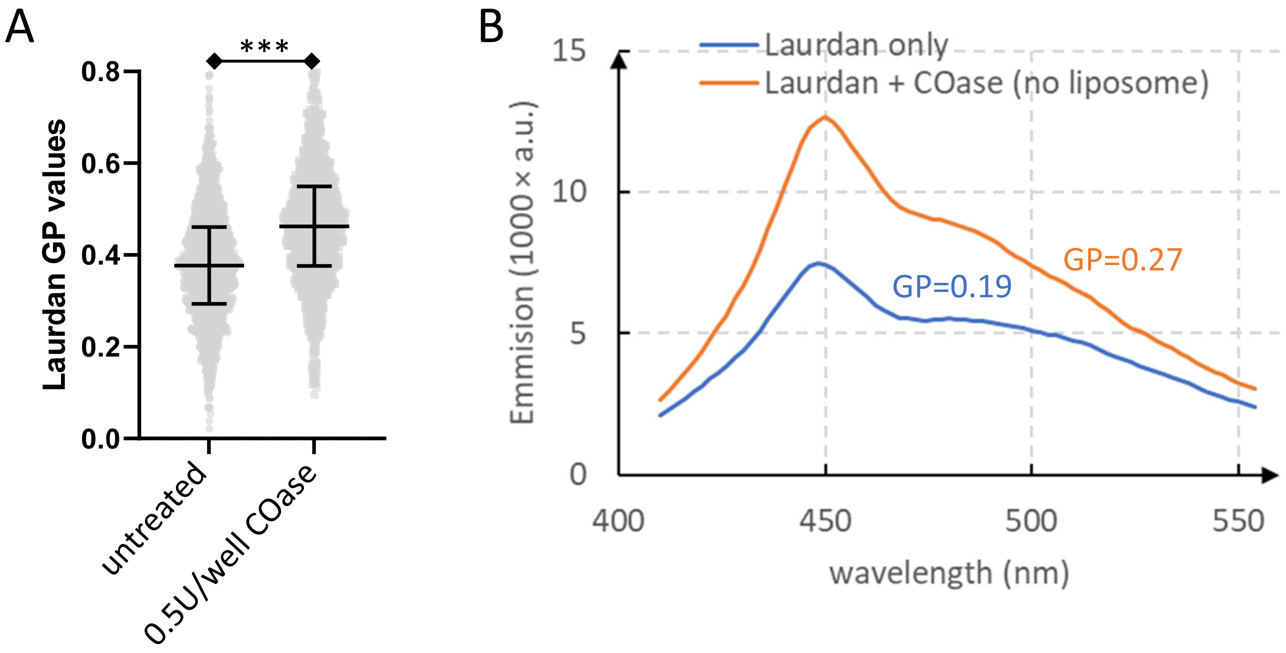

Figure S10. Lipid order measurement on COase treated pseudoviruses stained with Laurdan. Laurdan GP value distributions for control viruses and viruses treated with 0.5U of Coase (A) and Laurdan signal changes and GP increases with the addition of 0.5U COase (B). Statistics analysis was done by Kolmogorov–Smirnov test.

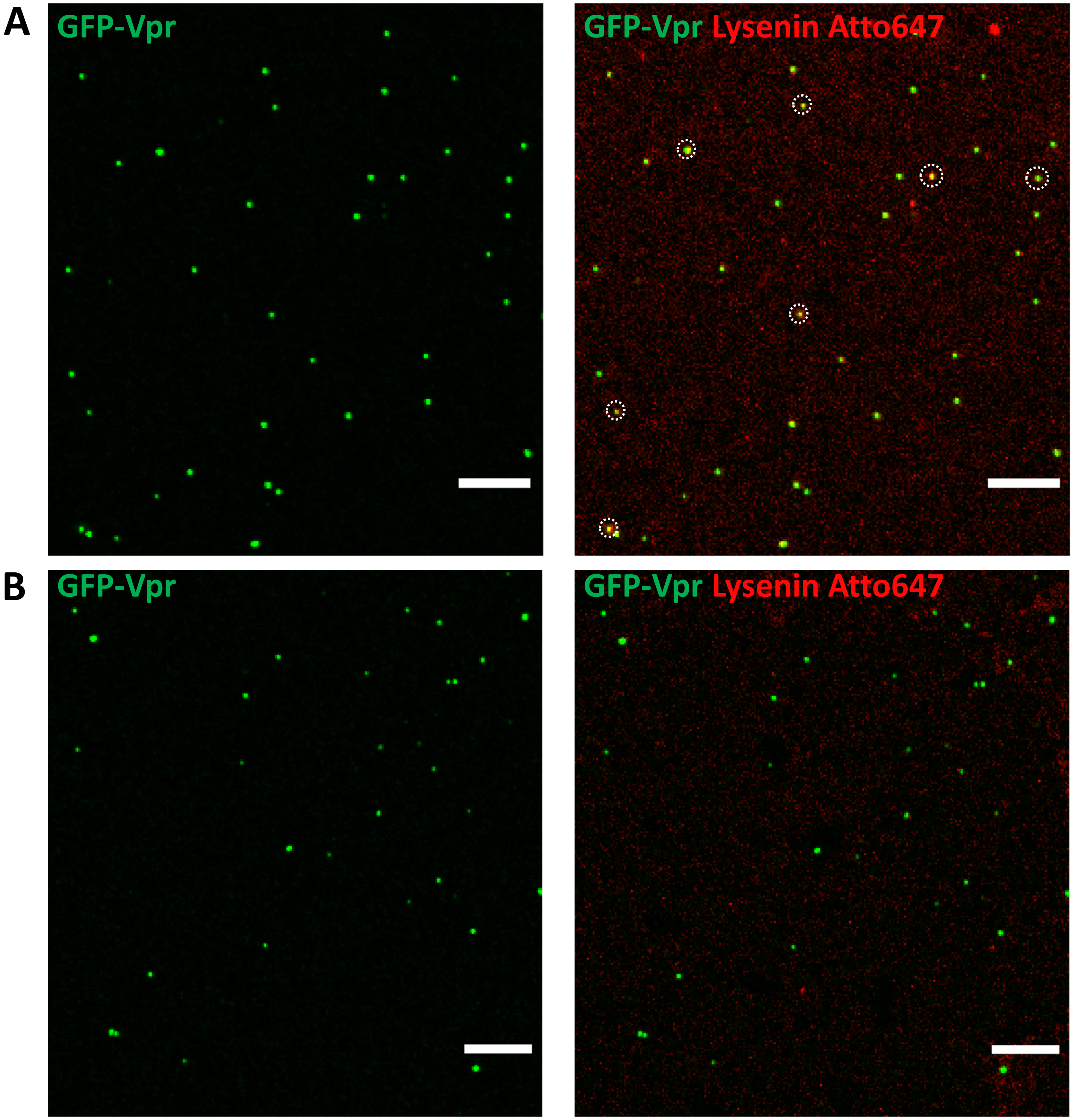

Figure S11. Lysenin Atto647 labeling of SMase treated pseudoviruses. Images of immobilized untreated pseudoviruses (A) and pseudoviruses treated with 0.05U SMase (B) labeled with GFP-Vpr (green) and stained with Lysenin Atto647 (red). The lack of colocalization in the green and red channels indicate successful hydrolytic cleavage of viral SM headgroups after SMase treatment. Dotted circles indicate colocalizing virus particles. Scale bar (5 μm).

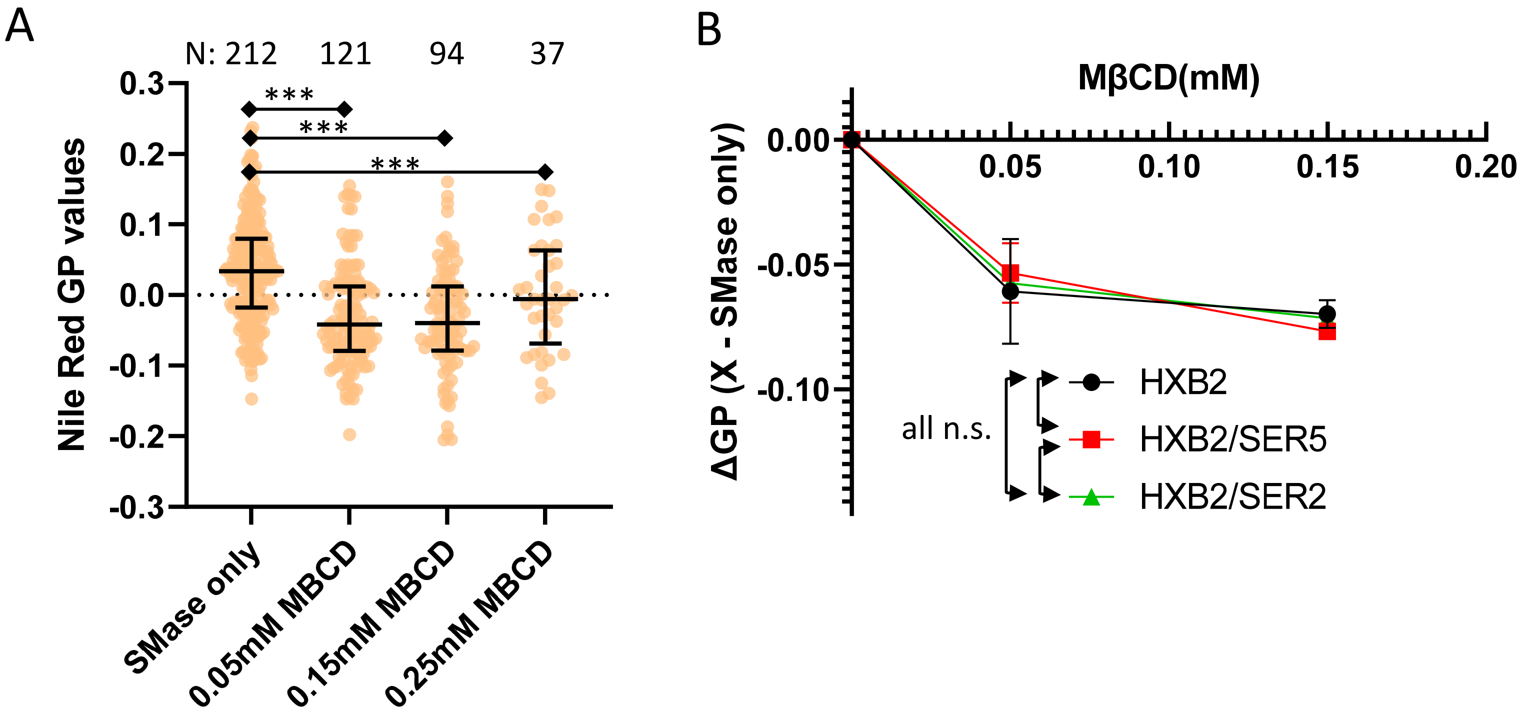

Figure S12. Assessing the effects of MβCD on viral lipid order on viruses pre-treated with SMase. (A) Nile Red staining of attached viruses. Horizontal black lines on the scatterplot represent 1st, 2nd, and 3rd quantiles. N, the number of viruses analyzed per experiment. (B) Means and standard deviations of ΔGP from 2 independent Nile Red measurements. Each virus was compared by 2-way ANOVA repeated measures. n.s., $p>0.05$; ***, $p<0.001$.
